# Can linezolid be validly measured in endotracheal aspiration in critically ill patients? A proof-of-concept trial

**DOI:** 10.1186/s40635-024-00630-x

**Published:** 2024-05-08

**Authors:** Diana Rebholz, Uwe Liebchen, Michael Paal, Michael Vogeser, Johannes Starp, Caroline Gräfe, Clara I. Brozat, Felix L. Happich, Katharina Habler, Christina Scharf

**Affiliations:** 1 Department of Anesthesiology, University Hospital, LMU Munich, Munich, Germany; 2 Institute of Laboratory Medicine, University Hospital, LMU Munich, Munich, Germany

**Keywords:** Linezolid, Critically ill patient, Endotracheal aspiration (ENTA), Therapeutic drug monitoring, Bronchoalveolar lavage (BAL)

## Abstract

**Background:**

Therapeutic drug monitoring (TDM) of anti-infectives such as linezolid is routinely performed in blood of intensive care unit (ICU) patients to optimize target attainment. However, the concentration at the site of infection is considered more important for a successful therapy. Until now, bronchoalveolar lavage (BAL) is the gold standard to measure intrapulmonary concentrations of anti-infective agents. However, it is an invasive method and unsuitable for regular TDM. The aim of this proof-of-concept study was to investigate whether it is possible to reliably determine the intrapulmonary concentration of linezolid from endotracheal aspiration (ENTA).

**Methods:**

Intubated ICU patients receiving 600 mg intravenous linezolid twice daily were examined in steady state. First, preliminary experiments were performed in six patients to investigate which patients are suitable for linezolid measurement in ENTA. In a second step, trough and peak linezolid concentrations of plasma and ENTA were determined in nine suitable patients.

**Results:**

Linezolid can validly be detected in ENTA with viscous texture and > 0.5 mL volume. The mean (SD) linezolid trough concentration was 2.02 (1.27) mg/L in plasma and 1.60 (1.36) mg/L in ENTA, resulting in a median lung penetration rate of 104%. The mean (SD) peak concentration in plasma and ENTA was 10.77 (5.93) and 4.74 (2.66) mg/L.

**Conclusions:**

Linezolid can validly be determined in ENTA with an adequate texture and volume. The penetration rate is comparable to already published BAL concentrations. This method might offer a simple and non-invasive method for TDM at the site of infection “lung”. Due to promising results of the feasibility study, comparison of ENTA and BAL in the same patient should be investigated in a further trial.

## Background

Infections are a major challenge in intensive care units. 54% of patients in intensive care units have a confirmed infection and 70% receive at least one antibiotic [1]. Furthermore, the 30-day mortality rate for septic shock in Germany is as high as 30% despite different approaches to optimize therapy [2]. Anti-infective therapy has, therefore, a crucial role in the treatment of patients with severe infections. However, the dosing of anti-infectives is more difficult in intensive care unit (ICU) patients, as they have altered pharmacokinetics due to profoundly altered pathophysiological processes [3, 4]. Routine therapeutic drug monitoring (TDM) of especially antibiotics in ICU patients is recommended to reduce the risk of under- or overdosing, to maximize the efficacy, and to minimize toxicity [5–7], hopefully leading to a higher rate of target attainment.

Linezolid is regularly used in ICU patients with pneumonia or skin and soft tissue infections caused by gram-positive bacteria, such as methicillin-resistant Staphylococcus aureus (MRSA) or vancomycin-resistant enterococci (VRE) [8]. Large inter- and intra-individual differences in blood concentrations of linezolid were observed, leading to a complex dosing strategy [9]. Furthermore, the concentration in the blood does not automatically indicate the concentration at the site of infection, which is hypothesized to be more relevant for a successful therapy [10]. It has been shown that patients with acute respiratory distress syndrome or pneumonia have significantly lower linezolid blood concentrations than patients with non-pulmonary infections [11, 12]. Moreover, linezolid has been described to have a high penetration rate into the lung tissue [13]. It is not yet understood which linezolid concentration should be aimed for in the blood to achieve adequate concentrations in the lungs.

The gold standard for determining the intrapulmonary concentration of anti-infectives such as linezolid is the measurement in the epithelial lining fluid (ELF) obtained during bronchoscopy with bronchoalveolar lavage (BAL) [14]. However, bronchoscopy is an invasive procedure and not suitable for routine TDM as it, i.e., leads to a loss of positive end-expiratory pressure (PEEP) [15]. In addition, the secretion in the BAL is diluted with saline, so the TDM methods must be developed with a considerably lower calibration range and the actual concentration is calculated using the urea method, which is also prone to error [16].

The aim of this proof-of-concept study was to evaluate whether a reliable quantitative linezolid determination from endotracheal aspiration (ENTA) is possible. If possible, this would provide a simple and non-invasive method for TDM of linezolid at the site of infection “lung”, which would be an important step towards personalized dosing of anti-infectives.

## Methods

### Study design

This prospective, monocentric, proof-of-concept study was conducted at the LMU Hospital in Munich in two anesthesiologic ICUs. The local review board (project number 22-0490) gave ethics approval. The study was registered in the German Register for Clinical Studies (DRKS00030870).

Intubated, adult ICU patients receiving 600 mg intravenously linezolid twice daily, were included. Informed consent for the study was obtained from the patients or their legal representative. First, preliminary experiments were performed to define and develop the conditions and methodology for a TDM of linezolid from ENTA. Thereafter, the trough and peak concentration of linezolid in ENTA and plasma were determined in patients considered suitable for ENTA sampling and linezolid TDM. All demographic and clinical data were obtained from the patients’ medical records.

### Sampling, processing, and measurement

The steady-state trough and peak levels were used to compare the concentrations in ENTA and plasma. The ENTA was obtained in a standardized manner. Whenever possible, the patient was not suctioned for 1 h before sampling and no inhalatives were administered 1 h before sampling. A closed suction system (14Ch, suction depth 54 cm) with the same negative pressure (− 20 mmHg) was always used. Linezolid was quantified from plasma that was obtained from arterial blood and ENTA. The linezolid concentration in plasma was determined using an established routine isotope-dilution liquid chromatography–tandem mass spectrometry (ID LC–MS/MS) method with a lower limit of quantification of 0.125 mg/L [17]. To qualify ENTA for linezolid measurement with the very same ID LC–MS/MS method, the viscous secretion had first to be liquefied. The aspirate was diluted 1:2 (v/v) with Proteinase-K solution (1 mg/mL Proteinase-K in 50 mM Tris–HCL, 200 mM CaCl2, total pH 7.5), and after short vortexing, the mixture was incubated in a water bath at 37 °C for 20 min. Preliminary experiments confirmed stability of linezolid in ENTA at 37 °C for 90 min (deviation <  ± 15%). The plasma and liquified ENTA were stably stored up to 1 month at -80 °C until ID LC–MS/MS analysis.

### Statistical analysis

Statistical analysis was performed using IBM SPSS Statistics (version 29.0.0.0). Normal distribution was tested using the Shapiro–Wilk test. Values were expressed as mean and standard deviation (SD) or as median and interquartile range (IQR). Pearson correlation was used to correlate trough levels of ENTA and plasma.

## Results

Preliminary experiments were performed in six patients to define and develop the conditions and methodology for a TDM from ENTA. In five out of six patients, the ENTA volume was ≤ 0.5 mL and/ or had a liquid texture, suggesting the hypothesis of condensate accumulation. Linezolid could not or not validly be detected in these samples. Table 1 displays the ENTA volume and texture as well as the measured trough concentration in the ENTA and the site of infection.

**Table 1:** ENTA volume, texture, trough concentration, and site of infection of preliminary experiments

Patient	Volume (mL)	Texture	Trough concentration ENTA (mg/L)	Site of Infection
1	0.4	Viscous	< 0.125	Abdomen
2	0.3	Liquid	1.26	Abdomen
3	0.2	Liquid	< 0.125	Extremity
4	0.8	Viscous	0.92	Abdomen + lung
5	0.5	Liquid	< 0.125	Abdomen
6	1.0	Liquid	< 0.125	Extremity

Thereafter, only patients with a viscous aspirate > 0.5 mL were included and a total of nine patients was observed for the proof-of-concept trial. Seven patients had a proven pulmonary infection confirmed by either image morphology or microbial detection. Patients 2, 4, 5, 6, 7, 8, and 9 were treated with inhalatives (71% Amphotericin B, 71% Ipratropium bromid + Salbutamol, 57% Sodium chloride 3%, 14% Colistin). The reason for admission to the ICU was in descending order: respiratory failure/ARDS (*n* = 4), sepsis/septic shock (*n* = 3), polytrauma (*n* = 1), liver transplantation (*n* = 1). Table 2 shows further patient characteristics.

**Table 2:** Patient characteristics

Patient characteristics	*n* (%)	Mean (SD)	Median (IQR)
Number of patients	9 (100)		
Male	8 (88.9)		
Age (years)		59 (17)	
Body size (cm)		183 (10)	
Body weight (kg)			86 (25.5)
SAPS II at start linezolid therapy		63 (15)	
APACHE II on admission to ICU		26 (8)	
Length of stay in ICU (days)		47 (18)	
Indication linezolid: E. faecalis	5 (55.6)		
Indication linezolid: calculated	4 (44.4)		
Proven pulmonary infection	7 (77.8)		
28-day mortality	2 (22.2)		

*SAPS* simplified acute physiology score, *APACHE* acute physiology and chronic health evaluation

Linezolid trough and peak concentrations were measured in plasma and ENTA that are displayed in Table 3. In patients 1, 3, and 6, sufficient viscous secretion was used to determine trough concentrations of plasma and ENTA at two timepoints.

**Table 3:** Linezolid trough and peak concentrations in plasma and ENTA

Patient	ENTA volume	Trough conc. plasma (mg/L)	Trough conc. ENTA (mg/L)	Ratio ENTA/plasma (%)	Peak conc. plasma (mg/L)	Peak conc. ENTA (mg/L)
1	0.8/0.7	3.78 / 3.22	0.92 / 0.34	24.3 / 10.6	15.59	2.82
2	0.9	0.05	0.48	960.0	3.39	2.80
3	3.2/1.3	2.94 / 3.15	2.42 / 4.72	82.3 / 149.8	7.45	4.52
4	1.5	0.31	0.32	103.2	5.41	1.36
5*	0.6	2.98	3.16	106.0	16.17	6.82
6	1.3/0.6	1.21 / 1.88	1.38 / 1.08	114.1 / 57.4	9.37	3.24
7	2.3	0.52	0.58	111.5	4.42	3.88
8	2.0	2.49	2.62	105.2	17.92	9.04
9*	0.9	1.69	1.22	72.2	17.19	8.20
Mean (SD)		2.02 (1.27)	1.60 (1.36)		10.77 (5.93)	4.74 (2.66)
Median (IQR)				104.2 (43.6)		

*No proven pneumonia

**Fig. 1:**
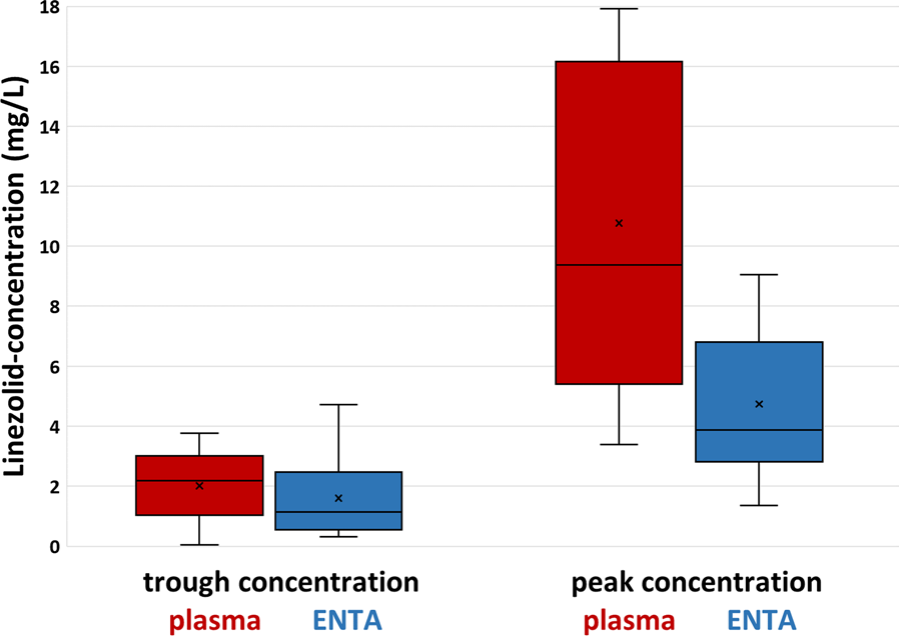
Linezolid trough and peak concentration in plasma and ENTA. The boxes of the boxplots represent the interquartile range (IQR), the line the median, the cross the mean and the whiskers the minimum and maximum value. The red boxplots represent the trough and peak concentrations in plasma and the blue boxplots represent the trough concentrations in ENTA

The mean (SD) linezolid trough levels were 2.02 (1.27) mg/L in plasma and 1.60 (1.36) mg/L in ENTA. The median ENTA/plasma ratio was 104.2%. Pearson correlation showed a significant and moderate correlation between plasma and ENTA trough levels (*r* = 0.52, *p* < 0.05). The mean (SD) linezolid peak concentrations were 10.77 (5.93) mg/L in plasma and 4.74 (2.66) mg/L in ENTA with a median penetration rate of 47.7%. Figure 1 illustrates the linezolid trough and peak concentrations in plasma and ENTA.

**Fig. 2:**
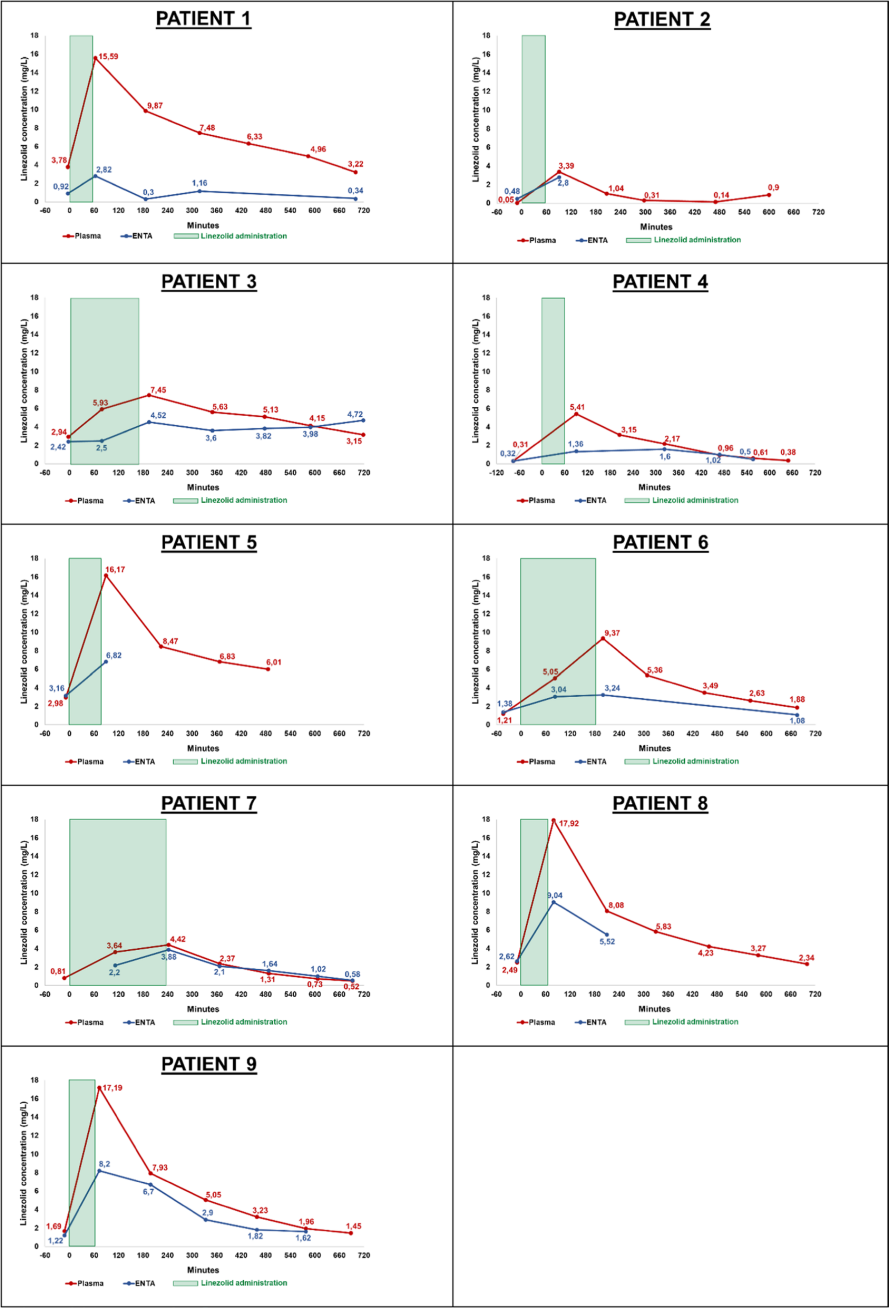
Linezolid concentration in ENTA and plasma over the 12-h dosing interval. The red line represents the concentration of linezolid in the plasma and the blue line the concentration in the ENTA. The green box shows the administration time of linezolid. Only ENTA values with a volume > 0.5 mL and a viscous texture are shown

If sufficient secretions could be aspirated, further linezolid measurements were carried out in the ENTA and in the blood. Figure 2 shows the linezolid concentration of the 9 patients in the ENTA (blue) and plasma (red) over the 12-h dosing interval.

## Discussion

The concentration of antibiotics in the blood does not automatically indicate the concentration at the site of infection. Linezolid is often used in critically ill patients with pneumonia due to a postulated high penetration rate and is associated with a better outcome than the treatment with vancomycin [13, 18]. The measurement of linezolid in patients´ lung, if the suffer from pneumonia, seems, therefore, promising [14, 19]. The high rate of *E. faecalis* in our collective might be caused by the high number of immunosuppressed patients after severe infections with prolonged intensive care stay or organ transplantation. In other hospitals, the detection of, e.g., pneumococci, is to be expected.

Measuring linezolid concentrations in endotracheal aspirate is a potential new, simpler, and non-invasive method compared to BAL. It would be less time-consuming and does not require additional equipment, such as a bronchoscope. If the suctioning is performed with a closed system, the patient is not exposed to any loss of PEEP or risk of infection [15]. However, the suction depth, the thickness of the catheter and a potential biofilm formation in the endotracheal tubes could have an influence on the quality of the sample and the included linezolid concentration. In contrast, a standardized BAL also has different limitations: the dwell time plays a central role in the percentage of the drug that is transferred into the aspirate [20]. Furthermore, the high dilution with NaCl leads to very low drug concentrations with the necessity of reevaluating the calibration range. The transfer factor is typically estimated using the urea method, which, as already mentioned, is only an estimate [21, 22].

The preliminary measurements taught us that validly measurement of linezolid in the ENTA is possible in samples with sufficient volume (> 0.5 mL) and viscous texture. If there is too little liquid secretion, it is possible that only the condensation water from the ventilation tube is drawn off, and therefore, no valid measurement is possible. Patients with pneumonia mostly have viscous pulmonary secretions and the linezolid concentration in the lung is especially of interest in those patients. Linezolid trough concentrations in ENTA were shown to be comparable to plasma concentrations in appropriate patients, as confirmed by a moderate correlation coefficient of 0.52. In fact, 58% of the trough levels were even higher in ENTA than in plasma, which has already been observed in the BAL, demonstrating the good penetration of linezolid into the lung and indicating that our method might be valid for non-invasive measurement of linezolid in the critically ill [19, 23].

Although also peak concentrations were measured in plasma and ENTA and further concentrations in the dosing interval, no reliable interpretation can be made due to the lack of knowledge about the penetration velocity in patients with intermitted dosing. To improve the interpretation of the data, a pharmacokinetic model would be beneficial in the future. Significant differences were observed in the lung penetration rate of linezolid (e.g., patient 1 with a very low penetration rate). No specific cause can be named for this, although large inter-individual differences in the linezolid blood concentration are already known and the reasons for this are at least partially transferable to the lungs [9, 12]. This again demonstrates the need for TDM of linezolid not only in the blood but also at the site of infection to identify patients like patient 1 with low penetration rates and to enable the target range to be achieved in these patients as well [3, 21, 24].

Only four published studies were identified that investigated the intrapulmonary trough concentration of intravenously administered linezolid in critically ill patients using the gold standard “BAL”. This already shows that the current data situation is very limited. Boselli et al*.* studied the intrapulmonary concentration of linezolid in 16 patients with ventilator-associated pneumonia. The mean linezolid penetration into ELF was 104% [25]. In another study, Boselli et al*.* investigated the alveolar diffusion of linezolid during continuous linezolid administration in 12 patients with a median (IQR) linezolid penetration rate of 97% [19]. De Pascale et al*.* received a median penetration rate of 80% in seven critically ill obese patients [14]. Finally, Wu et al*.* studied 23 patients with sepsis and was able to show a penetration rate of 112% [10]. The median penetration rate into the ELF in these four studies is 101%. Based on the available data, all studies demonstrate a low variability of the penetration rate.

We observed a median ENTA/plasma ratio of the trough concentrations of 104%. The penetration rate measured in our study appears to be comparable to the median penetration rate measured in the ELF in the above-mentioned studies. It should be noted that a direct comparison is not possible as different patients were used. This allows the hypothesis to be derived that the measurement of linezolid in the ENTA might be a valid method for the future. To test this hypothesis, the measurement of linezolid in ENTA and BAL in the same patient at the same time is pending. The primary goal of examining whether linezolid can be measured in endotracheal aspiration in patients with viscous secretions was thus achieved.

This proof-of-concept trial has several limitations. Only nine patients were included and, therefore, less patients as in the published studies where linezolid was measured in the ELF. However, the goal of the proof of concept study was to verify if and when a measurement of linezolid in the ENTA is possible at all. This goal was achieved by including nine selected and valuable patients. Since the speed of linezolid penetration into the lungs is not yet known, it is unclear whether the linezolid peak levels in the blood corresponds to the peak level in the lungs when measured at the same time. A pharmacokinetic model could, therefore, be helpful. The penetration of linezolid into pneumonic areas may be limited, as they are less perfused under the assumption of the Euler–Liljestrand reflex. A BAL directly from this area might contribute to an even more precise determination of the concentration at the site of infection. Finally, linezolid in the lung was only determined in ENTA and not additionally from the BAL in the same patient. A direct comparison of the concentrations is not possible yet. The next step should be to directly compare the concentration in the ENTA with the concentration in the BAL in the same patient at the same time. Thereafter, ENTA can perhaps be used as a non-invasive standard method in the future.

## Conclusion

Linezolid can be reliably quantified in ENTA with adequate texture and volume. This might offer a new and simple way to determine the concentration of linezolid for TDM at the site of infection “lung”, allowing even more personalized and targeted therapy for suitable and selected patients. In the future, validation of the method with the gold standard "BAL" for measuring the intrapulmonary concentration is recommended.

## Data Availability

Linezolid was determined at more measurement times than the trough level, but no further conclusions can be drawn from the descriptive analysis.

## Abbreviations

BAL: Bronchoalveolar lavage
ELF: Epithelial lining fluid
ENTA: Endotracheal aspirates
ICU: Intensive care unit
ID LC–MS/MS: Isotope dilution liquid chromatography–tandem mass spectrometry
PEEP: Positive end-expiratory pressure
TDM: Therapeutic drug monitoring

## References

[1] Vincent JL (2020). Prevalence and outcomes of infection among patients in intensive care units in 2017. JAMA.

[2] Bauer M (2021). Mortality in sepsis and septic shock in Germany. Results of a systematic review and meta-analysis. Anaesthesist.

[3] Abdul-Aziz MH (2020). Antimicrobial therapeutic drug monitoring in critically ill adult patients: a position paper. Intensive Care Med.

[4] Roberts JA, Lipman J (2009). Pharmacokinetic issues for antibiotics in the critically ill patient. Crit Care Med.

[5] Evans L (2021). Surviving sepsis campaign: international guidelines for management of sepsis and septic shock 2021. Intensive Care Med.

[6] Roberts JA (2014). Individualised antibiotic dosing for patients who are critically ill: challenges and potential solutions. Lancet Infect Dis.

[7] Lin B (2022). Expert consensus statement on therapeutic drug monitoring and individualization of linezolid. Front Public Health.

[8] Bodmann KF, Grabein B, Kresken M (2020). S2k guideline "Calculated parenteral initial treatment of bacterial infections in adults—update 2018", 2nd updated version: foreword. GMS Infect Dis.

[9] Zoller M (2014). Variability of linezolid concentrations after standard dosing in critically ill patients: a prospective observational study. Crit Care.

[10] Wu C (2022). Pharmacokinetic/pharmacodynamic parameters of linezolid in the epithelial lining fluid of patients with sepsis. J Clin Pharmacol.

[11] Taubert M (2016). Predictors of inadequate linezolid concentrations after standard dosing in critically ill patients. Antimicrob Agents Chemother.

[12] Zoller M (2022). Serum linezolid concentrations are reduced in critically ill patients with pulmonary infections: a prospective observational study. J Crit Care.

[13] Roger C, Roberts JA, Muller L (2018). Clinical pharmacokinetics and pharmacodynamics of oxazolidinones. Clin Pharmacokinet.

[14] De Pascale G (2015). Linezolid plasma and intrapulmonary concentrations in critically ill obese patients with ventilator-associated pneumonia: intermittent vs continuous administration. Intensive Care Med.

[15] American Association for Respiratory, C. AARC Clinical Practice Guidelines. Endotracheal suctioning of mechanically ventilated patients with artificial airways 2010. Respir Care. 2010; 55: 758–764. 20507660

[16] Paal M (2021). Target site pharmacokinetics of meropenem: measurement in human explanted lung tissue by bronchoalveolar lavage, microdialysis and homogenized lung tissue. Antimicrob Agents Chemother.

[17] Paal M, Zoller M, Schuster C, Vogeser M, Schutze G (2018). Simultaneous quantification of cefepime, meropenem, ciprofloxacin, moxifloxacin, linezolid and piperacillin in human serum using an isotope-dilution HPLC-MS/MS method. J Pharm Biomed Anal.

[18] Kato H (2021). Meta-analysis of vancomycin versus linezolid in pneumonia with proven methicillin-resistant *Staphylococcus aureus*. J Glob Antimicrob Resist.

[19] Boselli E (2012). Alveolar diffusion and pharmacokinetics of linezolid administered in continuous infusion to critically ill patients with ventilator-associated pneumonia. J Antimicrob Chemother.

[20] Baughman RP (2007). Technical aspects of bronchoalveolar lavage: recommendations for a standard procedure. Semin Respir Crit Care Med.

[21] Kiem S, Schentag JJ (2014). Interpretation of epithelial lining fluid concentrations of antibiotics against methicillin resistant *Staphylococcus aureus*. Infect Chemother.

[22] Rodvold KA, Yoo L, George JM (2011). Penetration of anti-infective agents into pulmonary epithelial lining fluid: focus on antifungal, antitubercular and miscellaneous anti-infective agents. Clin Pharmacokinet.

[23] Kiem S, Schentag JJ (2008). Interpretation of antibiotic concentration ratios measured in epithelial lining fluid. Antimicrob Agents Chemother.

[24] Pea F, Cojutti PG, Baraldo M (2017). A 10-year experience of therapeutic drug monitoring (TDM) of linezolid in a hospital-wide population of patients receiving conventional dosing: is there enough evidence for suggesting TDM in the majority of patients?. Basic Clin Pharmacol Toxicol.

[25] Boselli E (2005). Pharmacokinetics and intrapulmonary concentrations of linezolid administered to critically ill patients with ventilator-associated pneumonia. Crit Care Med.

